# Effect of tinnitus on sound localization ability in patients with normal hearing

**DOI:** 10.1016/j.bjorl.2023.01.003

**Published:** 2023-01-30

**Authors:** Yue Long, Wei Wang, Jiao Liu, Ke Liu, Shusheng Gong

**Affiliations:** a Department of Otolaryngology-Head and Neck Surgery, Beijing Friendship Hospital, Capital Medical University, Beijing, China; b Clinical Center for Hearing Loss, Capital Medical University, Beijing, China

**Keywords:** Sound source localization, Tinnitus, Tinnitus handicap inventory

## Abstract

**Objectives:**

To determine whether tinnitus negatively impacts the accuracy of sound source localization in participants with normal hearing.

**Methods:**

Seventy-five participants with tinnitus and 74 without tinnitus were enrolled in this study. The accuracy of sound source discrimination on the horizontal plane was compared between the two participant groups. The test equipment consisted of 37 loudspeakers arranged in a 180° arc facing forward with 5° intervals between them. The stimuli were pure tones of 0.25, 0.5, 1, 2, 4, and 8 kHz at 50 dB SPL. The stimuli were divided into three groups: low frequency (LF: 0.25, 0.5, and 1 kHz), 2 kHz, and high frequency (HF: 4 and 8 kHz) stimuli.

**Results:**

The Root Mean Square Error (RMSE) score of all the stimuli in the tinnitus group was significantly higher than that in the control group (13.45 ± 3.34 vs. 11.44 ± 2.56, *p* = 4.115, *t* < 0.001). The RMSE scores at LF, 2 kHz, and HF were significantly higher in the tinnitus group than those in the control group (LF: 11.66 ± 3.62 vs. 10.04 ± 3.13, *t* = 2.918, *p* = 0.004; 2 kHz: 16.63 ± 5.45 vs. 14.43 ± 4.52, *t* = 2.690, *p* = 0.008; HF: 13.42 ± 4.74 vs. 11.14 ± 3.68, *t* = 3.292, *p* = 0.001). Thus, the accuracy of sound source discrimination in participants with tinnitus was significantly worse than that in those without tinnitus, despite the stimuli frequency. There was no difference in the ability to localize the sound of the matched frequency and other frequencies (12.86 ± 6.29 vs. 13.87 ± 3.14, *t* = 1.204, *p* = 0.236). Additionally, there was no correlation observed between the loudness of tinnitus and RMSE scores (*r* = 0.096, *p* = 0.434), and the Tinnitus Handicap Inventory (THI) and RMSE scores (*r* = −0.056, *p* = 0.648).

**Conclusions:**

Our present data suggest that tinnitus negatively impacted sound source localization accuracy, even when participants had normal hearing. The matched pitch and loudness and the impact of tinnitus on patients’ daily lives were not related to the sound source localization ability.

**Level of evidence:**

4.

## Highlights

• Tinnitus is a common disorder that affects hearing and social interactions.
• Patients with tinnitus have reported hearing difficulties in noisy environments.
• Tinnitus negatively impacts sound source localization in those with normal hearing.
• It might also affect the processing of brain cues for sound source discrimination.

## Introduction

Subjective tinnitus is a conscious perception of an auditory sensation in the absence of any physical sound stimulation. It is typically described as ringing, buzzing, whooshing, or humming, and occasionally as a musical melody.^1^ Tinnitus is a common disorder with prevalence ranging from 10% to 25%.^2^ However, only 2%–3% of patients with tinnitus are severely impaired by it.^3^ Tinnitus is a multidimensional disorder that extends far beyond hearing.^3^, ^4^ Many patients with tinnitus report problems with attentional control, anxiety, and interference with social interaction.^5^, ^6^ In addition, patients with tinnitus often report hearing difficulties, especially in noisy environments.^7^, ^8^ We hypothesized whether this hearing difficulty is related to hearing loss or the defect in shifting attention to interest speakers.

The ability to locate sound sources helps listeners orient toward sound sources of interest and selectively attend to the talker.^9^ Sound source localization mainly relies on three cues: Interaural Time Differences (ITD) and Interaural Level Differences (ILD) cues for horizontal localization and spectral-shape cues for vertical and front/back localization. Localization of sound sources is a complex process in the human brain. The spatial cues come from both ears and are analyzed in specific brainstem pathways. ITD cues are processed mainly in the Medial Superior Olive (MSO), ILD cues mainly in the Lateral Superior Olive (LSO), and spectral-shape cues in the Dorsal Cochlear Nucleus (DCN).^10^ Integration of these inputs in the primary auditory cortex, planum temporal, and posterior superior temporal gyrus represent the sound source locations,^11^ and the intact auditory pathway is indispensable for normal sound localization. A previous study focused on the auditory localization of participants with unilateral tinnitus, suggesting that tinnitus-related activity in localization-sensitive areas might interfere with localization cues, resulting in degraded localization performance.^12^

Currently, literature on sound localization in patients with tinnitus is scarce. Hyvärinen et al.^12^ conducted sound source discrimination tasks in horizontal and vertical planes. They recruited participants with unilateral tinnitus and hearing loss at high frequencies, matched hearing loss, and normal hearing. Their results indicated that tinnitus might degrade sound localization ability, but this effect was strongly related to hearing loss. In another study, An et al.^13^ only included participants with normal hearing and found that participants with tinnitus performed significantly worse than those without tinnitus. In this study, the results of localization were presented as an error score, which was calculated by scoring 1 point for each 30 degrees of difference between the target speaker and the response speaker. However, this scoring method is uncommon. Furthermore, as the error score increased with the number of recognition errors, regardless of the total number of stimulations, playing each speaker five times possibly amplified the errors in the tinnitus group. In addition, 30° intervals between the loudspeakers limited the localization accuracy to be measured with fine spatial resolution.^14^ Thus, we hypothesized that reducing the interval between speakers and using a more general scoring method may yield more convincing results.

In the present study, an array with a 5° angle of separation between the speakers was used, and only participants with normal hearing were recruited. The Root-Mean-Square Error (RMSE) score was calculated as the mean target response difference. This study aimed to determine whether tinnitus had a negative impact on the accuracy of sound source discrimination in participants with normal hearing.

## Methods

### Participants

**Figure 1:**
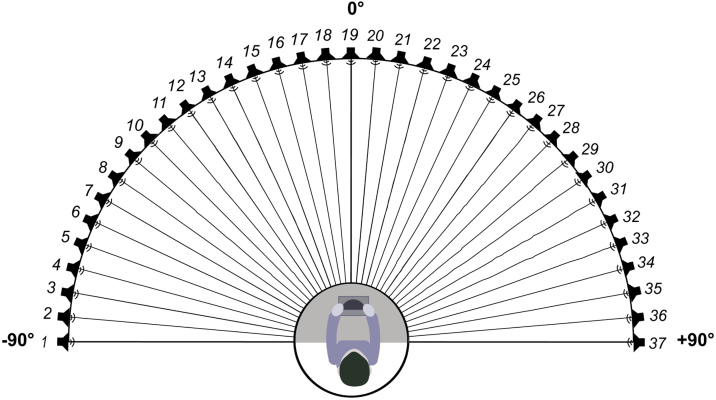
Schematic representation of the speaker placement for stimulus presentation.

Participants with normal hearing with and without tinnitus were enrolled in the study. Participants with tinnitus were recruited in the ENT outpatient department of the participating hospital from April 2021 to March 2022. The inclusion criteria for this group of patients were: 1) Subjective tinnitus, 2) Persistent tinnitus, 3) Duration ≥ 3 months, and 4) Hearing threshold ≤ 25 dB HL at all frequencies (0.25–8 kHz) in both ears (according to the World Health Organization classification).^15^ Participants in the control group had normal hearing and no tinnitus. They were recruited through advertisements, and the vast majority were students from Capital Medical University or staff from Beijing Friendship Hospital; they were not related to the participants with tinnitus. The inclusion criteria for the control group were as follows: 1) No tinnitus and 2) Hearing threshold ≤ 25 dB HL at all frequencies (0.25–8 kHz) in both ears. The exclusion criteria for both groups were as follows: 1) Objective tinnitus, 2) Difference in bilateral hearing threshold > 10 dB HL, 3) Air-bone gap > 10 dB HL, 4) History of hearing loss or dizziness, and 5) Diagnosis of depression or anxiety. The participants in the tinnitus group were slightly older than those in the control group (*t* = 4.336, *p* < 0.001). Fig. 1 shows that the hearing thresholds of the two groups were similar, except at 4 kHz in the right ear (12.40 ± 6.00 vs. 10.27 ± 5.79, *t* = 2.205, *p* = 0.029) and 1 kHz in the left ear (10.60 ± 4.57 vs. 8.85 ± 5.33, *t* = 2.147, *p* = 0.033). In the tinnitus group, the average duration of tinnitus was 21.44 ± 36.92 months. Thirty-four participants had unilateral tinnitus (16 and 18 in the right and left ears, respectively), and 41 had bilateral tinnitus.

This study was approved by the ethics committee of the participating hospital (2021-P2-004-01). The study procedures adhered to the tenets of the Declaration of Helsinki, and the participants signed an informed consent form before recruitment.

### Sound source discrimination task

The sound source discrimination task was performed in a soundproof room (LSsx2021-21270). Thirty-seven loudspeakers were set in a 180° arc, 5° apart from each other (Fig. 1). The speakers were 1.2 m away from the participant and at the height of the participant’s external auditory canal. The stimuli were pure tones of 0.25, 0.5, 1, 2, 4, and 8 kHz at 50 dB SPL for 2 s. In the experimental paradigm, we randomly presented 0.25, 0.5, 2, 4, and 8 kHz stimuli six times and 1 kHz stimuli seven times from the 37 loudspeakers. Each speaker played only once, and the participants were instructed to face directly ahead until the stimuli stopped and to indicate the speaker number (1–37) on a touchscreen. The experimenter monitored the participants’ head movements away from 0° azimuth while the stimuli were playing, and no feedback was provided after each response. The formal test began after participants had responded 10 times and were familiar with the process. The Root-Mean-Square Error (RMSE) score was calculated as the mean target response difference, and lower scores indicated greater accuracy.

### Tinnitus matching

Tinnitus matching included pitch and loudness using audiometers (Madsen Astera, Otometrics, USA) with headphones (ME70, Otometrics, USA). A two-alternative forced choice method was used in the process.^16^ First, the test ear was given a pair of pure-tone signals starting with multiples of 1 kHz, and the patient was asked to identify which one was closer to the tinnitus frequency. Once a pair of frequencies were identified, the frequency resolution increased, getting closer to the tinnitus frequency (the finest frequency was 1/48 octave). Then, the intensities of the matched pitch were incrementally increased in 5 dB steps, starting with an intensity below the hearing threshold and then gradually increasing or decreasing in 1 dB steps until the loudness of tinnitus was matched.

### Tinnitus handicap inventory

The Tinnitus Handicap Inventory (THI) is a self-report questionnaire comprising 25 items that reflect the impact of tinnitus on daily life. Each question can be answered with “yes, sometimes, or no”, with each response counting for 4, 2, or 0 points, respectively. The total score is graded on five scales: slight (0–16), mild (18–36), moderate (38–56), severe (58–76), and catastrophic (78–100).^17^ It is a widely used assessment tool that is sensitive to tinnitus severity.^18^

### Statistics

Continuous distribution variables are expressed as means and standard deviations. An independent samples *t*-test was performed to evaluate the differences between the two groups. One-way Analysis of Variance (ANOVA) and Least Significant Difference test (LSD, post hoc testing) were used to compare differences between frequencies. A paired-sample *t*-test was performed to evaluate differences in the RMSE scores of all the stimuli and matched frequency. The Pearson correlation test was used to detect the relationship between the RMSE score and matched loudness and the relationship between the RMSE and THI scores. Multiple linear regression was used to control for the confounding factors of age to explore the difference in auditory localization between the two groups. Statistical analyses were performed using SPSS version 25 (IBM Corp., Armonk, NY, USA), and *p-*values < 0.05 were considered statistically significant.

## Results

**Figure 2:**
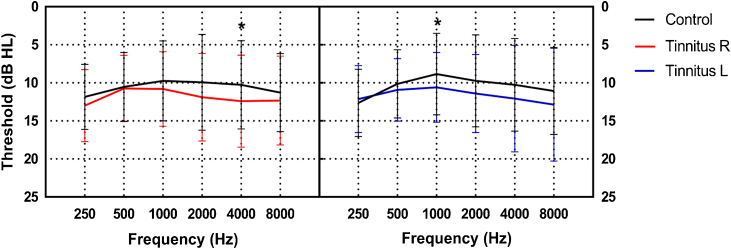
Comparison of the mean hearing thresholds at each frequency between the tinnitus and control groups (error bars denote one standard deviation). The black lines indicate the mean hearing threshold of the control group. The red and blue lines indicate the mean hearing threshold of the right and left ears of the tinnitus group, respectively (*p < 0.05).

Seventy-five participants with tinnitus (30 men and 45 women, 20–55 years old, 35.28 ± 8.91 years) and 74 without tinnitus (24 men and 50 women, 17–51 years old, 29.22 ± 8.15 years) were enrolled in the study. Participants with tinnitus were recruited in the ENT outpatient department of the participating hospital from April 2021 to March 2022. Fig. 2 shows that the hearing thresholds of the two groups were similar, except at 4 kHz in the right ear (12.40 ± 6.00 vs. 10.27 ± 5.79, *t* = 2.205, *p* = 0.029) and 1 kHz in the left ear (10.60 ± 4.57 vs. 8.85 ± 5.33, *t* = 2.147, *p* = 0.033). In the tinnitus group, the average duration of tinnitus was 21.44 ± 36.92 months.

**Figure 3:**
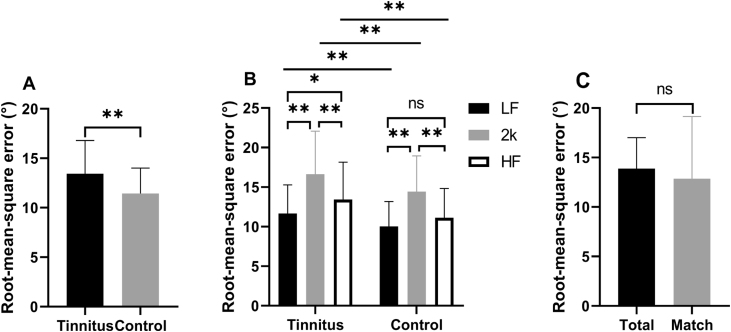
RMSE scores of the participants. (A) The RMSE scores for all the stimuli in the tinnitus and the control groups. (B) The two groups’ RMSE scores at LF, 2 kHz, and HF. (C) Comparison of RMSE score of all the stimuli and the matched pitch in the tinnitus group. LF, stimuli with 0.25, 0.5, and 1 kHz; 2 kHz stimulus with 2 kHz; HF, stimuli with 4 and 8 kHz; *p < 0.05, **p < 0.01, ns not significant.

The RMSE scores of the control and tinnitus groups were 11.44 ± 2.56 and 13.45 ± 3.34, respectively. The control group localized the sound sources more accurately than the tinnitus group (*p* = 4.115, *t* < 0.001; Fig. 3A). The difference between the two groups persisted when the confounding factor of age was controlled (RMSE: *b* = 1.238, *p* = 0.012; age: *b* = 0.127, *p* < 0.001). Since the localization of low-frequency (<1500 Hz) and high-frequency (>3000 Hz) sounds mainly depends on ITD and ILD cues, respectively,^19^, ^20^ the stimuli were divided into three groups: low-frequency (LF: 0.25, 0.5, and 1 kHz), 2 kHz, and high-frequency (HF: 4 and 8 kHz). In the tinnitus group, the RMSE score at 2 kHz was higher than that at LF and HF, and the RMSE score at HF was higher than that at LF. In the control group, the RMSE score at 2 kHz was higher than that at LF and HF; however, the difference between the RMSE scores at HF and LF was insignificant. The participants in both groups had the worst sound localization precision at 2 kHz. In addition, participants with tinnitus performed worse than those without tinnitus in localizing LF, 2 kHz, and HF stimuli (detailed data are shown in Table 1 and Fig. 3B).

**Table 1:** The RMSE scores at LF, 2 kHz, and HF in the tinnitus and control groups.

	Tinnitus	Control	*t*-Test		LSD (tinnitus)		LSD (control)	
			*t*	*p*	*t*	*p*	*t*	*p*
LF	11.66 ± 3.62	10.04 ± 3.13	2.918	0.004	‒	‒	‒	‒
2 kHz	16.63 ± 5.45	14.43 ± 4.52	2.690	0.008	4.972	<0.001	4.386	<0.001
HF	13.42 ± 4.74	11.14 ± 3.68	3.292	0.001	3.205	<0.001	3.289	<0.001
LF	‒	‒	‒	‒	1.767	0.021	1.097	0.082

RMSE, Root Mean Square Error; LF, stimuli at 0.25, 0.5, and 1 kHz; 2 kHz, stimulus at 2 kHz; HF, stimuli at 4 and 8 kHz; *t*-test, Independent sample *t*-test; LSD, least significant difference method. The LSD shows the results compared to the above row.

Forty-one participants in the tinnitus group heard a tinnitus pitch close to the stimuli (±1/6 octave). We evaluated the difference in RMSE scores for all the stimuli and the matched frequency and did not find any significant difference (13.87 ± 3.14 vs. 12.86 ± 6.29, *t* = 1.204 *p* = 0.236) (Fig. 3C). In addition, there was no correlation between the loudness of tinnitus and RMSE scores (*r* = 0.096, *p* = 0.434).

Seventy participants in the tinnitus group completed the THI questionnaire. The numbers of participants graded on the slight, mild, moderate, severe, and catastrophic scales were 13, 18, 19, 14, and 6, respectively. No correlation was observed between the THI and RMSE scores (*r* = −0.056, *p* = 0.648).

## Discussion

In this study, we performed a sound source discrimination test on the horizontal plane in a large group of participants with normal hearing with and without tinnitus. Our main finding was that sound source discrimination accuracy in participants with tinnitus was significantly worse than it was in those without tinnitus despite the frequency of stimuli. This observation was similar to those of previous studies.^12^, ^13^ The results indicated that tinnitus affects the processing of spatial cues in the auditory pathway because the localization of the sound source relies on precise transmission and computation through the auditory system.^21^

The participants in both groups had the worst precision in localizing the sound at 2 kHz. Sound localization in the horizontal plane mainly relies on ITD and ILD cues. The ITD results from the difference in the travel time from the source to the two ears. When the wavelength of the sound approaches the dimensions of the head, the available ITD cue increases as frequency decreases. ILD results from the head shadow effect, which mainly occurs in sounds with wavelengths shorter than the dimensions of the head.^10^ Our result was in line with the hypothesis that sound with an intermediate range of frequencies (roughly 1.5–3 kHz) had limited ITD and ILD cues available,^19^, ^20^ and indicated that the participants’ response was reliable.

An et al.^13^ suggested that the degraded localization performance in tinnitus is due to interference with ILD cues. However, as the frequency and loudness of individual tinnitus matched were not considered, this conclusion could not be drawn straightforwardly.^12^ In our study, the accuracy of localizing LF, 2 kHz, and HF stimuli in participants with tinnitus was worse than that in participants without tinnitus. This indicated that tinnitus affected the processing of both ITD and ILD in the auditory pathway.

The MSO principal cells exhibit robust sensitivity to ITD. Additionally, each principal neuron in the MSO receives excitatory inputs from the ipsilateral Anterior Ventral Cochlear Nuclei (AVCN) and trapezoid body from the contralateral AVCN. The MSO also receives inhibition projections from the medial and lateral nuclei of the trapezoid body (MNTB and LNTB, respectively).^10^, ^22^, ^23^ In contrast, the LSO contains neurons sensitive to ILD. Principal cells in the LSO receive excitatory inputs from the ipsilateral AVCN and inhibitory inputs from the contralateral AVCN via the MNTB.^24^ Then, ITD and ILD cues spread to the deep layers of the superior colliculus, which contains topographical maps of the auditory space.^21^ Convergent evidence indicates that the most common pathophysiologic mechanism of tinnitus is a net loss of inhibition,^25^, ^26^ which might interfere with the process of ILD and ITD cues, thereby affecting the ability of sound localization.

Our results showed that the ability to locate a sound similar to the matched pitch was consistent with the other frequencies in participants with tinnitus. Furthermore, no correlation was observed between tinnitus loudness and localization ability. This indicates that the degraded sound localization ability might not be directly related to interference with ILD cues. Investigations of the qualitative characteristics of tinnitus could not help predict the patients' ability to locate sound sources. This was in line with the previous guideline,^27^ in which pitch and loudness matching was not advised for diagnosing and treating tinnitus. In addition, psychoacoustic measures of tinnitus did not correlate well with self-reported measures of tinnitus annoyance.^28^ Therefore, self-report questionnaires were recommended to assess tinnitus severity and its impact on quality of life in patients.^29^ The THI is one of the most commonly used questionnaires to assess tinnitus impact worldwide.^30^ It can be divided into three subscales: 1) Functioning, which assesses limitations owing to tinnitus; 2) Emotions, which focus on emotional attitudes toward tinnitus; and 3) Catastrophic responses, which evaluate catastrophic thoughts about tinnitus. The global score provides an overall assessment of the extent to which tinnitus affects daily life. Our results showed no correlation between the THI and RMSE scores, indicating that the worsening sound source localization ability was not directly related to tinnitus distress experienced by patients.

Subjective tinnitus with normal hearing usually presents without any identifiable cause, leading to a lack of definitive treatment for relieving the symptoms. Recent studies have focused on various methods besides conventional audiograms to detect abnormal findings in patients with tinnitus who have normal hearing. Our study found that tinnitus patients with normal hearing performed worse in sound source localization than those without tinnitus, indicating that tinnitus affects the processing of ITD and ILD through the auditory system. Since the exact mechanisms of tinnitus are not fully understood, further investigation of the functional brain imaging and histocytochemical changes in the auditory pathways associated with ITD and ILD processing in patients with tinnitus or animal models can improve our understanding of tinnitus mechanisms.

This study had several limitations. First, the results only represented the ability to localize pure tones. Further study should be designed to investigate whether the same result would be found when the stimuli contain spectrum information. Second, as age-confounded comparisons between the two groups were made, age-matched participants should be enrolled in future studies. Third, although statistical significance was found between the two groups, the impact on individual life was unknown. Thus, the results regarding the localization ability of participants with tinnitus should be interpreted carefully and considered as a starting point for further studies.

## Conclusion

Our present data suggest that tinnitus negatively impacted the accuracy of sound source localization, even in participants with normal hearing. Furthermore, we found that tinnitus might affect ITD and ILD processing through the auditory pathway. Finally, the matched pitch, loudness, and impact of tinnitus on patients’ daily lives were unrelated to their ability to locate sound sources.

## Funding

This work was supported by National Key Scientific Research Project of China (2020YFC2005201).

## Conflicts of interest

The authors declare no conflicts of interest.
